# The Arabic Generalized Anxiety Disorder 2 (GAD-2): Psychometric evaluation among mothers of children with intellectual disabilities

**DOI:** 10.1080/19585969.2026.2650296

**Published:** 2026-05-05

**Authors:** Amira Mohammed Ali, Saeed A. Al-Dossary, Feten Fekih-Romdhane, Carlos Laranjeira, Haitham Khatatbeh, Heba Emad El-Gazar, Ahmad Ayed, Abdulmajeed A. Alkhamees, Musheer A. Aljaberi, Rasmieh Alamer, Annamaria Pakai, Mohamed Ali Zoromba

**Affiliations:** ^a^ Department of Psychiatric Nursing and Mental Health, Faculty of Nursing, Alexandria University, Alexandria, Egypt; ^b^ Department of Psychology, College of Education, University of Ha’il, Ha’il, Saudi Arabia; ^c^ Faculty of Medicine of Tunis, Tunis El Manar University, Tunis, Tunisia; ^d^ Department of Psychiatry Ibn Omrane, The Tunisian Center of Early Intervention in Psychosis, Razi Hospital, Tunis, Tunisia; ^e^ School of Health Sciences, Polytechnic University of Leiria, Leiria, Portugal; ^f^ Centre for Innovative Care and Health Technology (ciTechCare), Polytechnic University of Leiria, Leiria, Portugal; ^g^ Comprehensive Health Research Centre (CHRC), University of Évora, Évora, Portugal; ^h^ Faculty of Nursing, Yarmouk University, Irbid, Jordan; ^i^ Department of Nursing Administration, Faculty of Nursing, Port Said University, Port Said, Egypt; ^j^ Faculty of Nursing, Arab American University, Jenin, Palestine; ^k^ Department of Psychiatry, College of Medicine, Qassim University, Buraidah, Saudi Arabia; ^l^ Section Nursing Science, Department of Internal Medicine, Erasmus University Medical Center (Erasmus MC), Rotterdam, Netherlands; ^m^ Faculty of Health Sciences, Institute of Nursing Sciences, Basic Health Sciences and Health Visiting, University of Pécs, Pécs, Hungary; ^n^ Department of Nursing, College of Applied Medical Sciences, Prince Sattam Bin Abdulaziz University, Al-Kharj, Saudi Arabia; ^o^ Psychiatric and Mental Health Nursing Department, Mansoura University, Mansoura, Egypt

**Keywords:** Generalised Anxiety Disorder 2-item scale/GAD-2, psychometrics/validation, receiver operator characteristic curve/ROC/cut-off score/cut-off point, mothers/children with mental disabilities, low mood/stress/sleep/nightmares/physical health, Arabic/Saudi

## Abstract

**Introduction:**

Disruptive behaviours of children with intellectual disabilities predispose mothers to mental and physical morbidities, leading to caregiving burnout, lower childcare quality, and poor child progress.

**Methods:**

This cross-sectional study investigated the psychometrics of the Arabic version of the Generalised Anxiety Disorder 2-item scale (GAD-2) among 85 Saudi mothers of children with intellectual disabilities through latent variable model and receiver-operating characteristic curve analyses.

**Results:**

The unidimensional GAD-2 demonstrated good construct validity, invariance at the configural, metric, and scalar levels across age groups, and adequate convergent/divergent validity—It was negatively predicted by high mood and happiness and positively predicted by stress, and it mediated the effect of stress and happiness on depression. Its known-group validity was determined by elevated anxiety levels among mothers using psychotropic drugs. Two cut-offs (≥2.5 and ≥3.5) flagged the best trade-off between sensitivity and specificity for predicting low mood, poor sleep quality, nightmares, high stress, low general physical health, and willingness to join a psychological support program. The positive predictive value for the cut-off ≥3.5 was higher for all outcomes than that of the cut-off ≥2.5.

**Discussion:**

The GAD-2 is a valid and reliable tool, which at thresholds ≥3.5 can identify anxious mothers, aiding early diagnosis and intervention.

## Introduction

Anxiety is a feeling of non-specific fear and worry from anticipated danger (Lahoud et al. 2026). Generalised anxiety disorder (GAD) is a common disabling mental disorder that manifests primarily with serious pathological anxiety, with worries being chronic, excessive, and uncontrollable (Ahn et al. 2019; Luo et al. 2019; Byrd-Bredbenner et al. 2021). Worldwide, GAD has a one-year prevalence ranging from 2.4 to 18.2%. Anxiety disorders are ranked the sixth leading cause of global disability, accounting for more than 15% of the disability-adjusted life years (Luo et al. 2019; Lahoud et al. 2026). They are also associated with greater levels of depression and premature death secondary to high suicidality in burdened caregivers (Ali et al. 2024a, 2024b). Gender differences in the incidence of anxiety are well-documented, with higher occurrence among females than males (Byrd-Bredbenner et al. 2021). Anxiety is highly prevalent among the mothers of children with intellectual disabilities (Alblooshi et al. 2023; Lahoud et al. 2026), including those in the Arab region (Khamis 2007; Almansour et al. 2013).

Maternal anxiety is strongly linked to children’s everyday functioning, uncertainty, and concerns about their relationships with others. Particularly, children’s limited mental functioning and dependency on others in everyday activities (e.g., bathing, dressing, touching, and socializing) makes them vulnerable to sexual/financial exploitation and other forms of manipulation by others while being unable to recognise and report abusive situations (Gutowska 2022; Kamaludin et al. 2022). Apart from their risk for victimisation, mothers also fear that their children may encounter disproportionately negative experiences in the criminal justice system due to their impulsivity and disrupted behaviours, which put them at a great likelihood for inflicting sexual violence, homicide, theft, and arson among others (Sarrett and Ucar 2021; Giannouli 2022).

Financial drain is another source of anxiety in those families (Quashie et al. 2022; Del Bianco et al. 2024). While parents provide care to 60% of individuals with intellectual disabilities in Australia, the United Kingdom, and the United States (Davenport and Zolnikov 2022), social policies in different parts of the world rely on families regarding the financial responsibilities of their disabled children (Gutowska 2022; Moosa-Tayob and Risenga 2022; Quashie et al. 2022). Intensive family caregiving promotes socioeconomic inequalities, especially in socioeconomically disadvantaged groups (Quashie et al. 2022). Indeed, the mental health of mothers/parents and their children can be seriously endangered by socioeconomic factors such as low income, poverty, low parental education, and low-paid occupation (Del Bianco et al. 2024).

Sleep deprivation and poor sleep quality are common among the mothers of children with intellectual disabilities (Almeida et al. 2024; Ljubičić et al. 2025) due to extended night-time caregiving in response to their children’s symptoms of sleeplessness and disruptive behaviours (e.g., irritability) (Dietch et al. 2021; Almeida et al. 2024; Guner and Hayton 2024). Prolonged and frequent night-time awakening and activity evoke behavioural manifestations of circadian rhythm alteration, e.g., anxiety, depression, and poor quality of sleep (Alblooshi et al. 2023; Lahoud et al. 2026). At the neurochemical level, maternal sleep disturbances are associated with higher concentrations of evening cortisol, greater perceptions of psychological stress, and lower perceptions of self-esteem and happiness (Ljubičić et al. 2025). Meanwhile, disrupted sleep augments anxiety through a neurobiological mechanism involving dysconnectivity of the anterior default mode network (DMN, e.g., the medial prefrontal cortex) and posterior DMN (e.g., precuneus) (Shen et al. 2024).

Nightmares, a form of sleep disturbance that involves frightening dreams, commonly occur in the general population and to a greater extent in people with psychiatric conditions such as depression and anxiety (Dietch et al. 2021; Sayk et al. 2024). Nightmare content in clinically significant anxiety involves negatively toned themes, e.g., of death and abandonment (Mishra and Jain 2024). Nightmares are associated with cortical hyperarousal and distorted sleep architecture (longer Stage 1 sleep, longer Stage 2 sleep latency, and shorter slow-wave sleep duration), which functionally manifest with poor sleep efficiency and continuity, higher levels of distress, depression, anxiety, hyperarousal, and poor physical health (Simor et al. 2012; Sayk et al. 2024). Both cortical hyperarousal and anxiety are amenable to change by focused nightmare therapies such as imagery rehearsal, with no effect on sleep architecture or subjective sleep quality (Sayk et al. 2024).

Mothers of children with disabilities may be at risk for hospital admission either to accompany their children, who get frequently admitted, or as a result of their physical health collapse out of extensive caregiving (Graham et al. 2009). Maternal intensive care unit admissions occur following suicidal attempts aggravated by children’s mental and behavioural problems (Nakanishi et al. 2023). Therefore, healthcare professionals in different settings (e.g., critical care units, primary care, paediatric hospitals, psychiatric clinics, child rehabilitation centres, etc.) have particular interest in addressing the mental and physical health needs of those mothers as raising maternal self-confidence and empowerment aids in maximising the outcomes of childcare (Borglin et al. 2015). Calibrated screening tools may effectively identify mothers eligible for support programs and monitor their response to treatment (Ali et al. 2023a; Hlynsson and Carlbring 2024).

Diagnostic measures are core clinical tools and cost-effective alternatives for expensive or difficult to perform gold-standard tests (Habibzadeh et al. 2016). The Generalised Anxiety Disorder 7-item scale (GAD-7) closely follows the DSM-IV diagnostic criteria of GAD. Despite its relative brevity, frequent use measurement may be time consuming and impractical, leading to incomplete responses, especially as most test batteries combine multiple measures (Staples et al. 2019). Therefore, the shorter version, the GAD-2, is more desirable, and it has been widely used as a valid and reliable measure (Ahn et al. 2019; Luo et al. 2019; Byrd-Bredbenner et al. 2021; Veisy et al. 2021), with performance comparable to that of the GAD-7 in both healthy and clinical samples from different age groups (Staples et al. 2019; Byrd-Bredbenner et al. 2021; Veisy et al. 2021; Ibrahim et al. 2024; Macdonald-Gagnon et al. 2024). It expressed evident criterion validity by significantly correlating with high stress, poor sleep quality, substance abuse, suicidality, and poor mental health among American Veterans and Sudanese students (Ibrahim et al. 2024; Macdonald-Gagnon et al. 2024). It also correlated with lower academic performance, increased time spent in internet use for entertainment, low monthly expenditure, and less satisfaction with religious practices among students (Ibrahim et al. 2024). In experimental studies, the GAD-2 reflected changes in symptom severity and response to treatment (internet-based cognitive behavioural therapy) at efficiency greater than the Patient Health Questionnaire-2—an ultra-brief measure of depression—indicating significantly greater discriminative ability of the GAD-2 (Hlynsson and Carlbring 2024). Nonetheless, investigations from different cultural backgrounds (e.g., Chinese, Bangali, Chilian, etc.) show poor performance of the GAD-2 relative to longer versions (GAD-6 and GAD-3) (Ahn et al. 2019; Wang et al. 2024).

Within an Arab context, the GAD-2 significantly correlated with dysmorphic concerns, depression, perceived stress, and perceived stigma in Jordanian patients with general dermatologic diseases and cosmetic concerns. However, it did not significantly predict body dysmorphic disorder in this population at the commonly reported cut-off (≥ 3), suggesting limited psychometric properties of the Arabic GAD-2 (Murshidi et al. 2024). Global interest in the validation of psychometric measures among Arabs is growing because Arabic is spoken by more than 400 million native speakers in the Arab world, as well as by Arab immigrants and refugees, who exist in large numbers (ranging between 1.2 and 3.5% of the total population) in America, Europe, and Australia (Elshahat and Newbold 2021). Therefore, this study fills a gap in existing knowledge by exploring the psychometric properties of the Arabic version of the GAD-2 among mothers of children with intellectual disabilities—a rather distressed population.

## Materials and methods

### Design and participants

This cross-sectional study recruited a convenience sample of 85 mothers of children with intellectual disabilities. Data were collected electronically (between 22 October and 7 December 2023—when no more contributions were obtained) through an anonymous survey that was developed in Google forms and disseminated through Saudi special childcare centres as well as parent groups on Facebook and WhatsApp. Given that the GAD-2 comprises two items while the rest of the measures were single-item measures, the present sample, according to the rule of thumb (10 responses per each scale item, 10:1), can be sufficient for running the present model in the current study (Boateng et al. 2018).

The Research Ethics Committee of Ha’il University approved the data collection protocol (11/9/2023: H-2023-367). The participants were informed about the aim of the study. They were reassured about data anonymity, privacy, and voluntary participation. Those aged ≥18 years signed a digital informed consent before they were directed to the survey.

### Measures

Data were self-reported through an online questionnaire, which inquired about the sociodemographic characteristics of the mothers (age, marital status, and employment). Binary items (yes, no) explored their intake of formally prescribed psychotropic drugs and their desire to take part in a supportive program. One item explored their frequent experience of dreams/nightmares during the past month (Leggett et al. 2020). The questionnaire also included a set of brief measures, which examined other psychological factors.

The Generalised Anxiety Disorder 2-item scale (GAD-2) assesses the frequency of symptoms associated with anxiety (‘feeling nervous, anxious or on edge’ and ‘not being able to stop or control worrying’) during the past 2 weeks. Respondents rate their responses on a 4-point Likert scale, which ranges from 0 (not at all) to 3 (nearly every day). Thus, the total score of the GAD-2 ranges between 0 and 6 (Byrd-Bredbenner et al. 2021).

Mood was assessed by two measures. A single-item measure was used to assess overall mood: Rate your mood generally on a scale from 0 (very bad/the worst mood ever) to 10 (excellent/the best mood ever). Higher scores of this item reflect good mood (Gertler and Tate 2020). The second measure was the Patient Health Questionnaire 2-Item scale (PHQ-2). It comprises two items (‘little interest’ and ‘feeling down, depressed, or hopeless’), which assess the frequency of symptoms associated with depression during the past 2 weeks. Same as the GAD-2, respondents rate their responses on a 4-point Likert scale, which ranges from 0 (not at all) to 3 (nearly every day). Thus, the total PHQ-2 score ranges between 0 and 6 (Löwe et al. 2005; Ali et al. 2025a).

Fordyce’s single-item measure of happiness was used to assess happiness: Rate the extent to which you generally feel happy over the past week on a scale from 0 (very unhappy) to 10 (extremely) (Fordyce 1988). A single-item measure was used to assess stress: In the past year, how would you rate the amount of stress in your life (at home and at work/study)? It prompts the respondents to rate their overall stress on a 6-point scale, which ranges from 1 (no stress) to 6 (extreme stress) (Littman et al. 2006). Higher scores on both measures signify greater happiness and higher stress.

The single-item sleep quality scale (SQS) was used to assess sleep quality: Rate the quality of your sleep on a scale from 0 (very poor) to 10 (excellent) (Snyder et al. 2018). All the measures were translated into Arabic and back translated into English according to known standards to ensure adequate adaptation of the measures. No issues arose during the translation. The measures were preliminary tested before the commence of the study: to ensure the clarity of the measures the first author conducted a brief interview with five mothers in a childcare clinic and asked them to read the sentences and report their understanding. All the sentences were clearly understood.

### Statistical analysis

The characteristics of the sample were reported by suitable descriptive statistics, including the mean and standard deviation, median and interquartile range (Q1–Q3) for normally and non-normally distributed continuous variables, in order, as well as frequency and percentage for categorical variables.

Because confirmatory factor analysis of scales comprising less than four items yields a saturated model [Chi square index (*χ*^2^) and degree of freedom (*df*) = 0] (Ali et al. 2025b, 2025c), the construct validity of the GAD-2 was examined through a latent variable model, which comprised the PHQ-2, and measures of stress, mood, and happiness. Positive variables served the testing of divergent validity, while negative variables tested the convergent validity (at the global level)/concurrent validity. Criteria considered for good fit of the model included Comparative Fit Index (CFI) and Tucker–Lewis Index (TLI) equal to or >0.95, as well as root mean square error of approximation (RMSEA) and standardised root-mean-square residual (SRMR) <0.06 (Ali et al. 2021, 2022a).

Model stability was examined through multigroup analysis for the evaluation of measurement invariance across age groups—age was categorised according to median 40 (35–47 years) into two groups (up to 40 years and above 40); both groups were almost equal in size [*n* = 43 (50.6%) and 42 (49.4%), respectively]. Configural, metric, scalar/strong, and strict invariances were examined through four nested models to determine if (1) the unconstrained model is globally expressed in both groups, (2) item loadings are equivalent, (3) intercepts are equivalent, and (4) residuals/unexplained variance are equivalent across groups (Ali et al. 2025a, 2025d). The thresholds of three fit indices were consulted for determining significant changes in model fit across groups: ΔCFI and ΔTLI <0.20 and ΔRMSEA <0.15 (Ali et al. 2025b).

Because the intake of formally prescribed psychotropic drugs indirectly reflects having a psychiatric diagnosis, we examined known-group validity of the GAD-2 and the PHQ-2 by evaluating the differences in both measures between mothers receiving or not receiving psychotropic drugs through independent sample *t*-test. Welch’s *t*-test with adjusted DF was reported because of the assumption of equal variances was violated due to evident variations in group sizes (see the Results below).

Coefficient alpha and item-total correlations were used to examine the reliability of the GAD-2 and its convergent validity at the item level. The GAD-2 was used as a continuous variable in receiver-operating characteristic (ROC) curve analysis to predict low mood, poor sleep quality, poor physical health, stress, nightmares, and willingness to join a supportive intervention. Quartiles [the first (Q1, 25th percentile), second/median (Q2, 50th percentile), and third (Q3, 75th percentile)] are values that split a dataset into four equal parts. They are commonly used for setting cut-off thresholds in various contexts when no statistically tested performance benchmarks have been established. Research shows that quartiles are very close to statistically determined cut-offs (Sehestedt et al. 2012; Ali et al. 2024c, 2025b). The categorical form of all the outcome variables used in ROC analysis was obtained based on quartile scores (Supplementary Table 1, target groups are defined with ● ‘scores below the first quartile indicated low mood, poor sleep, low happiness, and poor physical health while stress scores above the third quartile indicated high stress’). Model fit was considered based on common fit indices [Area Under Curve (AUC), sensitivity, and specificity for all possible cut points, and the Youden index (sensitivity + specificity) – 1]. Ideally, they should be all closer to 1 (Ali et al. 2024a). Using highly probable cut-offs originating from ROC analysis, we created two categorical forms of the GAD-2 (≥2.5 and ≥3.5), and we used *χ*^2^ test to identify the positive predictive values (PPV) and the negative predictive values (NPV) of each cut-off. The analysis was conducted in SPSS version 22 and Amos version 26. The results were considered significant at probability levels below 0.05 in two-tailed tests.

## Results

The mothers were primarily middle-aged (mean age was 40.8 ± 8.3, range = 20–60 years), married (87.1%), housewives/unemployed (72.9%), and willing to receive psychological support (63.5%). Six mothers (7.1%) reported the intake of prescribed psychotropic drugs. The average scores of the PHQ-2, overall mood, perceived physical health, stress, and happiness were (3.2 ± 1.1), (5.7 ± 2.9), (5.6 ± 2.5), (4.0 ± 1.8), (3.5 ± 1.7), and (6.0 ± 2.9), respectively. Nightmares were reported by 22.4% of mothers.

**Figure 1.**
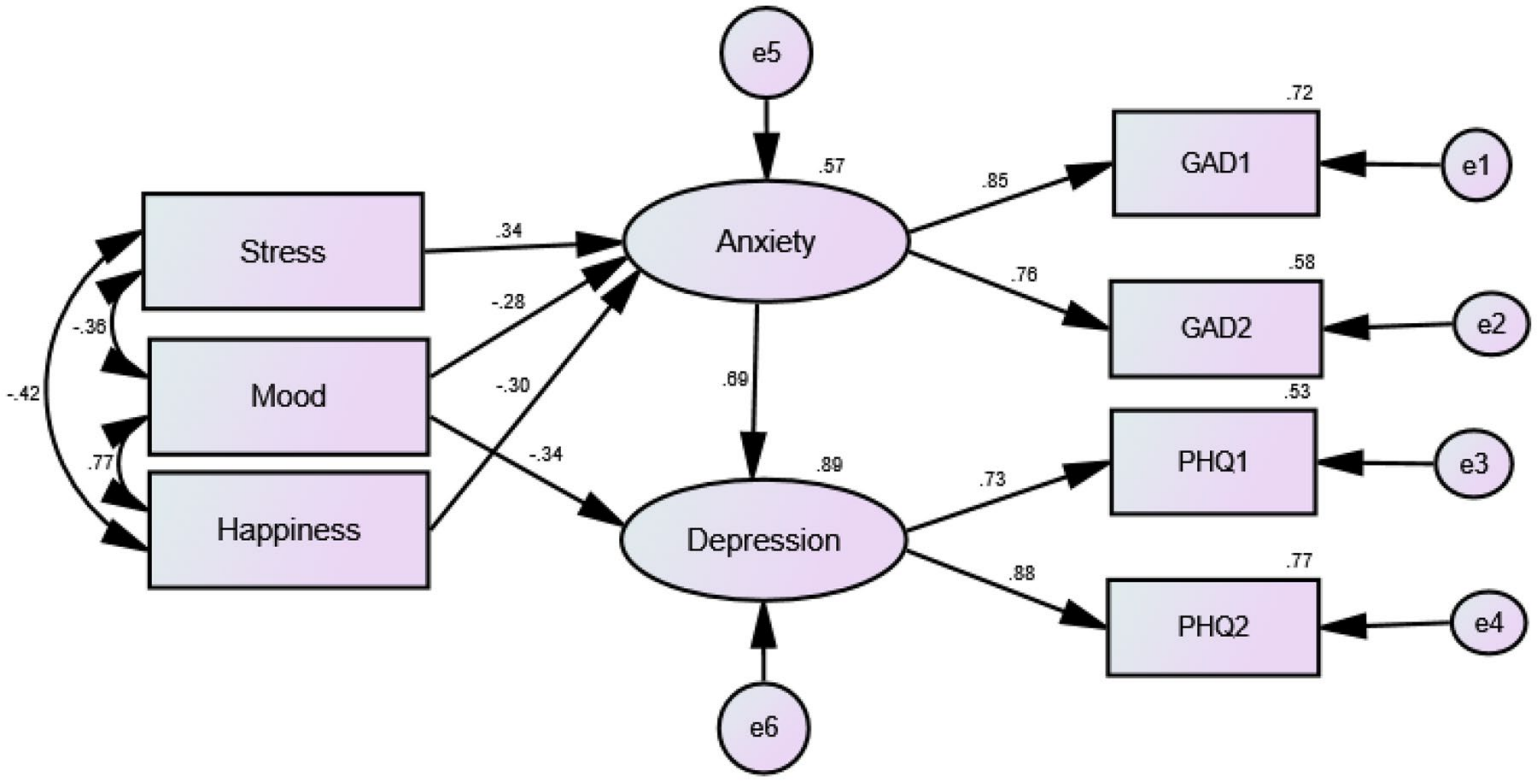
Latent variable model exploring the construct (convergent and divergent) validity of the Generalised Anxiety Disorder 2-item scale (GAD-2) among mothers of children with intellectual disabilities.

The latent variable model exhibited excellent fit (*χ*^2^ = 8.87, *df* = 11, *p* = 0.634, CMIN/DF = 0.81, CFI = 0.999, TLI = 0.999, RMSEA = 0.000, RMSEA 95% CI: 0.000 to 0.096, SRMR = 0.032). It predicted 57 and 89% of the variances in anxiety and depression. As shown in Figure 1, both items of the GAD-2 had significant loadings (>0.7) on the anxiety factor, supporting its construct validity. Anxiety as measured by the GAD-2 was directly predicted by mood, stress, and happiness; it significantly predicted depression and mediated the indirect effects of these variables on depression (*β* = −0.19, 0.24, and −0.21, respectively), which supports its convergent and divergent validity. The internal consistency of the GAD-2 was very good (Cronbach’s alpha = 0.81). The values of item-total correlations were also high (*r* = 0.68), suggesting strong convergent validity of the scale.

As indicated by multigroup analysis (Table 1), the model comprising the GAD-2 and the PHQ-2 demonstrated invariance at the configural, metric, and scalar levels across age groups—invariance at the strict level was not achieved.

**Table 1.** Measurement invariance of the Generalised Anxiety Disorder 2 (GAD-2) as indicated by multigroup analysis of structural equation model across age groups among Saudi mothers of children with intellectual disabilities (*N* = 85).

Invariance levels	*χ* ^2^	*df*	CMIN/*df*	*p*	Δ*χ*^2^	ΔDF	*p*(Δ*χ*^2^)	CFI	ΔCFI	TLI	ΔTLI	RMSEA	ΔRMSEA	SRMR
Configural	24.79	22	1.13	.307				0.993		0.989		0.039		0.0424
Metric	29.22	27	1.08	.350	4.43	5	0.490	0.983	0.010	0.978	0.011	0.031	0.008	0.0475
Scalar	38.46	33	1.17	.236	24.02	6	0.160	0.986	−0.003	0.983	−0.005	0.045	−0.014	0.0498
Strict	54.43	39	1.40	.051	15.01	4	0.005	0.952	0.034	0.948	0.035	0.069	−0.024	0.0747

*χ*^2^: chi-square; *df*: degree of freedom; CFI: comparative fit index; TLI: Tucker–Lewis index; RMSEA: root mean square error of approximation; CI: confidence interval; SRMR: standardised root mean residual.

The known-group validity of both measures was demonstrated by higher levels of anxiety and depression (6.33 ± 2.25, 6.83 ± 1.94) among mothers receiving psychotropic drugs (i.e., with diagnosed psychiatric disorders) relative to those not on psychiatric treatment (4.13 ± 1.74, 3.83 ± 1.61), *t*(5.47, 5.54) = 2.35, 3.69; *p* = 0.061, 0.012, respectively.

Table 2 and Supplementary Figure 1 present the results of ROC analysis. AUC values indicated moderate diagnostic accuracy of the GAD-2 for all outcomes, especially sleep quality, albeit the diagnostic accuracy for predicting the willingness to receive psychological support was the lowest among all as indicated by the lowest AUC (Supplementary Figure 1). The best balance between the highest values of both sensitivity and specificity in all the models was achieved at GAD-2 threshold of ≥2.5. According to this score, 81.2% of mothers may have clinically significant anxiety. Despite reductions in the Youden index at the GAD-2 threshold of ≥3.5, the positive predictive value (PPV) of the GAD-2 in all models was higher while the values of the negative predictive value (NPV) were largely similar to or higher than those noticed at the threshold ≥2.5. Based on GAD-2 ≥ 3.5 cut-off score, 61.2% of the mothers may have clinically significant anxiety. Implications for the use of this cut-off are discussed below.

**Table 2.** Cut-off scores of the Generalised Anxiety Disorder 2-item scale (GAD-2), along with goodness-of-fit indices associated with receiver-operating characteristic (ROC) curve analysis among mothers of children with intellectual disabilities.

	AUC	*SE*	*p*	AUC 95% CI	Cut-off	Sensitivity	Specificity	Youden index	PPV	NPV	Cut-off	Sensitivity	Specificity	Youden index	PPV	NPV
Mood	0.77	0.06	0.001	0.66 to 0.88	2.5	1.00	0.74	0.74	34.8	100.0	3.5	0.83	0.53	0.36	38.5	87.9
Sleep quality	0.79	0.06	0.001	0.68 to 0.90	2.5	1.00	0.77	0.77	21.7	100.0	3.5	0.93	0.54	0.47	26.9	97.0
Nightmares	0.72	0.06	0.004	0.59 to 0.84	2.5	0.95	0.77	0.72	26.1	93.8	3.5	0.84	0.55	0.39	30.8	90.9
General physical health	0.73	0.06	0.003	0.60 to 0.85	2.5	0.95	0.77	0.72	26.1	93.8	3.5	0.90	0.53	0.43	32.7	93.9
Stress	0.74	0.06	0.001	0.62 to 0.86	2.5	0.89	0.78	0.67	33.3	81.3	3.5	0.81	0.53	0.34	59.6	84.8
Joining support program	0.65	0.06	0.019	0.53 to 0.77	2.5	0.87	0. 71	0.58	68.1	56.3	3.5	0.69	0.48	0.17	71.2	48.5

AUC: area under the curve; *SE*: standard error; CI: confidence interval; PPV: positive predictive value; NPV: negative predictive value.

## Discussion

The GAD-2 demonstrates adequate psychometrics as a brief measure of GAD in Western cultures, but its robustness is questionable in Eastern cultures (Ahn et al. 2019; Murshidi et al. 2024; Wang et al. 2024). This study fills a gap in the literature by extending our understanding on the psychometric properties of the GAD-2 in a vulnerable Arab sample. The unidimensional GAD-2 is a reliable measure, which demonstrates adequate construct, convergent, divergent, and criterion validity as well as measurement invariance across age groups. It expressed moderate diagnostic accuracy at two cut-off points (≥2.5 and ≥3.5), with greater PPVs for the latter.

As shown in Figure 1, the standardised regression weights of the items of the GAD-2 were >0.7, denoting strong contribution of both items to the variance in their domain specific factor. Particularly, the fit of the model was exceptional in the absence of any error correlations (e.g., with items of the PHQ-2). This result indicates that both the GAD-2 and the PHQ-2 fulfil the assumption of local independence, which further supports the construct validity of both measures (Ali et al. 2022b, 2023b). Our report of the pure structures of the GAD-2 and the PHQ-2 is consistent with theoretical postulations, which indicate that lack of positive affect (e.g., expressed by the items of the PHQ-2 feeling ‘sad’ and ‘down and blue’) discriminates unipolar depression from anxiety disorders (Chin et al. 2019; Ali et al. 2024a). In this context, the GAD-2 and the PHQ-2 could distinctly detect GAD and depressive disorders.

Measurement invariance at the scalar level indicates measurement stability of the GAD-2 and the PHQ-2 in both younger and older mothers, i.e., mean differences in all the latent constructs in the model credibly detect all mean differences in the shared variance of their items. Research stresses the importance that measures of depression and anxiety are free of biases that are produced by age and gender differences (Astudillo-García et al. 2022). However, having a sample of mothers only precluded testing measurement equivalence across men and women. It was not possible to examine differences across marital status and employment groups because the exceptionally small numbers in some groups would bias test results (Ali et al. 2022b, 2025b).

Because of the overlap frequently reported between depression and anxiety (Ali et al. 2022b), we expected that the GAD-2 and the PHQ-2 would be strongly correlated. However, we did not initially hypothesise that anxiety would predict depression or mediate the effects of stress, mood, or happiness on depression as it was revealed later (Figure 1). These findings, which support the convergent validity of the GAD-2, are quite interesting as depression was evidently an outcome of anxiety and the interplay among anxiety and related factors such as stress and low positive wellbeing (lack of happiness). Accordingly, those with high GAD-2 scores may also require investigations of depressive disorders (Ahn et al. 2019). These findings may direct efforts aimed at improving maternal psychological health. Low mood, which was positively associated with psychological stress, was an evident feature of both anxiety and depression. However, both high stress and low positive well-being (happiness) were evident features of anxiety, but not depression. The latter resulted from a complex interplay of a variety of factors. Happiness as a key feature of positive well-being did not directly contribute to depression in our respondents, but it exerted an indirect effect through anxiety while it was strongly correlated with positive mood, which directly contributed to the score of the PHQ-2. Stress, which was negatively correlated with happiness, also did not directly contribute to depression but exerted an indirect effect through anxiety. Thus, the results suggest that promoting positive well-being through positive activities that are proven to support experiencing/regulating positive emotions may buffer stress, promote high mood, interrupt anxiety, and consequently remedy depression in those mothers. Positive emotion regulation activities operate by inducing structural changes that counteract shift from rewarding behaviour towards misery-avoidance in depression (Loonen and Ivanova 2016) as well as by upregulating thalamic GABA levels (Streeter et al. 2010), which are deficient in both depression and anxiety (Cryan and Kaupmann 2005).

In ROC analyses (Table 2), the GAD-2 as a criterion variable predicted six outcomes, which reflect debilitating psychological and physical problems (e.g., sleep disturbance and poor physical health). The values of the ACU fit index of all the models show moderate diagnostic accuracy of the GAD-2 (Table 2). The relation between sleep problems (e.g., insomnia and nightmares) and anxiety is well-established (Alblooshi et al. 2023; Shen et al. 2024; Lahoud et al. 2026). Same as the GAD-2 predicted stress (Table 2) and mediated the effect of stress on depression in our analyses (Figure 1), GAD has formerly mediated the effect of perceived stress on sleep quality (Huang et al. 2024). Moreover, nightmares interrupt sleep continuity and induce stress, depression, anxiety, hyperarousal, and poor physical health (Simor et al. 2012; Sayk et al. 2024). In line, the GAD-2 significantly predicted nightmares, poor sleep quality, and poor physical health in the present study. Taken together, the results suggest that the GAD-2 at the determined cut-off points may reflect higher likelihood of having depression, stress, sleep problems, poor physical health, and their possible interactions, necessitating the need to assess for the presence of these symptoms among GAD sufferers. In support of this conclusion, the GAD-2 predicted mothers’ willingness to engage in an intervention to support their mental well-being, which reflects perceived maternal burden that may be caused by their mental and physical symptoms.

It is worth mentioning that considerably higher PPVs occurred at the cut-off ≥3.5 while the NPVs were the same as or slightly lower than those occurring at the cut-off ≥2.5. High PPV and NPV indicate that persons who test positive are likely to have the disease and those who test negative do not likely have the disease, respectively (Ahn et al. 2019). In our analysis, sensitivity and specificity fit indices were reported for multiple cut-off scores, which did not include a GAD-2 score of ≥3. Unlike our findings, a recent meta-analysis of the optimal cut-offs of the GAD-2 revealed that the pooled sensitivity, specificity, and diagnostic odds ratio (0.80, 0.82, and 17.81, respectively) are the highest at the GAD-2 cut-off of 3 (Luo et al. 2019). Nonetheless, investigations of anxiety among American students through the GAD-7, GAD-2, GAD-Mini—two items revealed by iterative confirmatory principal components analysis of the GAD-7 stratified by sex—indicate that three quarters of males and two-thirds of females scored below cut-points that screen for GAD (Byrd-Bredbenner et al. 2021). Similarly, 60% of Korean adults were not actual anxiety disorder patients based on the GAD-2 cut-off score of 3 (Ahn et al. 2019). Likewise, the Arabic GAD-2 at the cut-off 3 did not predict body dysmorphic disorder among Jordanian dermatological patients (Murshidi et al. 2024). Single cut-offs originate from dichotomising continuous variables based on responses from specific samples, and they may not be generalisable, especially with lack of supporting biological evidence as numerous levels of the trait under concern are ignored, which does not apply to real-world scenarios (e.g., slightly ill and very ill) (Losciale et al. 2024). Therefore, we concluded that the cut-off ≥3.5 of the Arabic GAD-2 is likely the most suitable for the detection of GAD in this sample, and those scoring ≥3.5 may benefit from clinician-facilitated interviews to confirm the diagnosis of GAD. Given the high cost of clinical diagnostic procedures, the cut-off ≥3.5 may serve a paramount importance by identifying those who are highly prone to GAD (61.2%)—considerably fewer than those identified at the lower GAD-2 ≥ 2.5 score (81.2%).

The results of this study expand the literature on the psychometric properties of the GAD-2 by including a vulnerable population from the Arab world. The results have implications for research and practice; they support the construct validity of the GAD-2. The study also provides two cut-offs, which may be useful for predicting various symptoms of psychological distress (low mood, poor sleep, stress, and poor physical health). Employing a dual cut-off approach, which uses both a lower and upper threshold, has many advantages over a single cut-off. It allows appropriate clinical surveillance and more accurate and contextually relevant classifications by identifying individuals who are high-performing and not too low (i.e., severely sick as well as patients who are neither too sick nor too healthy). This would support a flexible approach to decision-making (Perno and Bertoli 2006; Caleyachetty et al. 2021). For example, those with moderate symptoms would receive early diagnosis and timely targeted interventions in resource-rich contexts. On the other hand, treatments are meant to have more immediate and visible impact in resource-limited settings (e.g., in most Arab countries), and they are likely to be directed to more severe rather than moderate conditions (Ali et al. 2025a). As such, mothers with a GAD-2 score ≥3.5 should benefit from advanced assessment for psychiatric morbidities (GAD, depression, and sleep disorders). The GAD-2 may be used to monitor response to supportive interventions aimed at improving maternal sleep quality as well as maternal mental and physical health. Nonetheless, the findings should be interpreted with caution because the study has many limitations.

We admit that the sample is relatively small and not representative, which may threaten the generalisability of the findings. This study examined the predictivity of the GAD-2 for outcomes that are frequently checked in clinical practice and research, and all the measures have been proved valid and reliable (e.g., PHQ-2). Nevertheless, admitting that not many mothers may complete the survey because of their enormous involvement in childcare, most outcomes were detected by single-item measures. It is not possible to reflect on the homogeneity of single-item measures in terms of reliability or construct validity since these measures do not tap different aspects of the underlying latent trait. However, single-item measures are becoming widely used in different disciplines as they are ultra-brief, and they undergo many other types of validity tests that focus on global constructs (e.g., convergent, divergent, discriminant, concurrent, predictive, and criterion). The literature also documents robust performance of these measures in psychology, marketing, and health relative to specific multiple-item measures (Hoeppner et al. 2011; Song et al. 2023). Given that multiple single-item measures were used in the study, and their associations are consistent with those reported in the literature, it may be possible that their adverse impact as single-item measures on our tests were not major. Moreover, some details of the detected outcomes were lacking, e.g., frequency of nightmares and if sleep quality was an effect of nightmares or involvement in caregiving at night-time. We also did not apply gold standards for identifying GAD, i.e., by not including other measures of anxiety, either lengthy measures or diagnostic interviews. Accordingly, future validation studies of the Arabic GAD-2 are recommended to use diagnostic interviews or longer anxiety inventories to ensure a confirmed diagnosis and more accurate diagnostic accuracy of the measure. The cross-sectional design, convenience sample, online survey, and inclusion of mothers from only one Arab country may have resulted in a sample with sociodemographic and clinical characteristics that do not represent the target population. Investigations of anxiety among Arabs show differences among countries, with specific patterns characterising its epidemiology in the Arab region. Cultural, social, religious, and economic factors are documented to be interwoven with the effect of young age, female gender, and acculturative stress (Tanios et al. 2009; AlAzzam et al. 2021; Tineo et al. 2021; El-Refaay et al. 2025). These factors may likely affect participants’ experience, expression, and interpretation of the symptoms addressed by the GAD-2. Therefore, further testing of the Arabic GAD-2 in larger samples from many Arab countries may be necessary to evaluate the interaction of various cultural factors with the GAD-2.

## Conclusion

The internal consistency, measurement invariance, as well as the construct, convergent, divergent, known-group, and criterion validity of the Arabic version of the GAD-2 support its usefulness as a measure of anxiety. At the cut-off points ≥2.5 and ≥3.5, the GAD-2 may indirectly reflect low general mood, low positive affect, high stress, poor sleep quality, nightmares, and poor physical health. The higher GAD-2 cut-off (≥3.5) may be more credible and cost-effective than the cut-off ≥2.5, especially in under-resourced settings. Replication of the study in samples from different Arab countries and in relevance to lengthy measures of anxiety or diagnostic interviews may confirm the findings.

## Supplementary Material

Supplementary_Figure1_GAD_2.docx

Supplementary_Table1_GAD_2.docx

## Data Availability

The dataset used and/or analysed during the current study is available from the corresponding author on reasonable request.
