# Supplementary material for: The Arabic Generalized Anxiety Disorder 2 (GAD-2): Psychometric evaluation among mothers of children with intellectual disabilities
Source: Dialogues Clin Neurosci. 2026 May 5;28(1):21–31. doi: 10.1080/19585969.2026.2650296 (PMC13148081; doi:10.1080/19585969.2026.2650296)
Supplement: Supplementary_Figure1_GAD_2.docx [file TDCN_A_2650296_SM7251.docx]

| 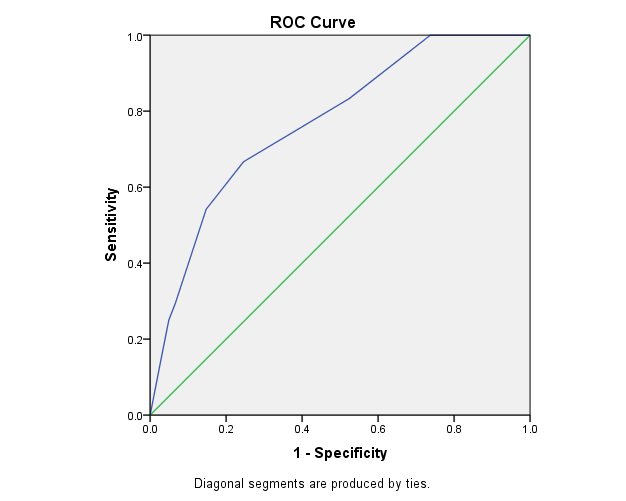   1. Mood | 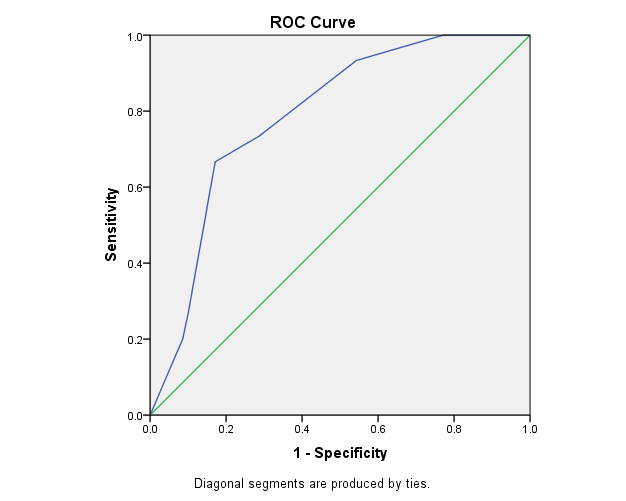   1. Sleep quality |
| --- | --- |
| 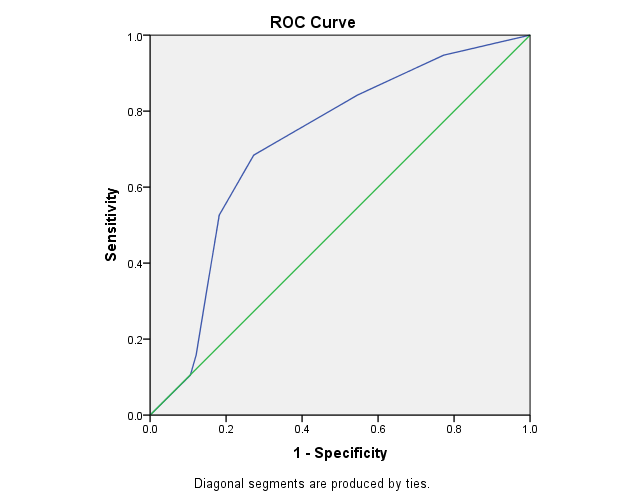   1. Nightmares | 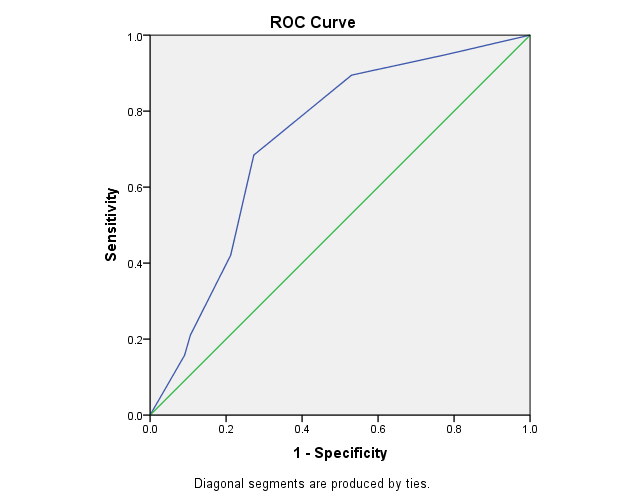   1. General physical health |
| 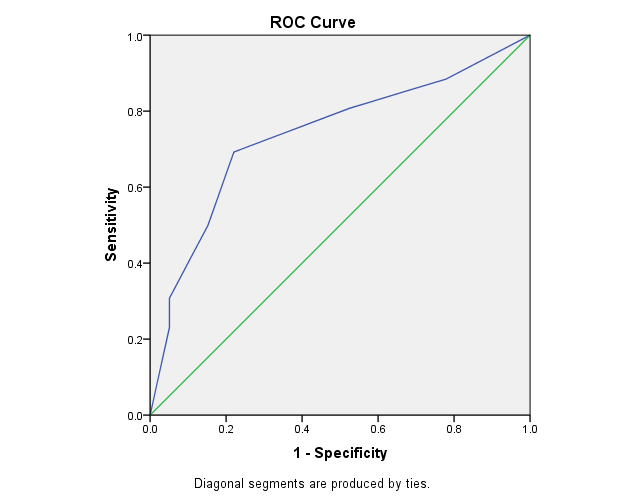   1. Stress | 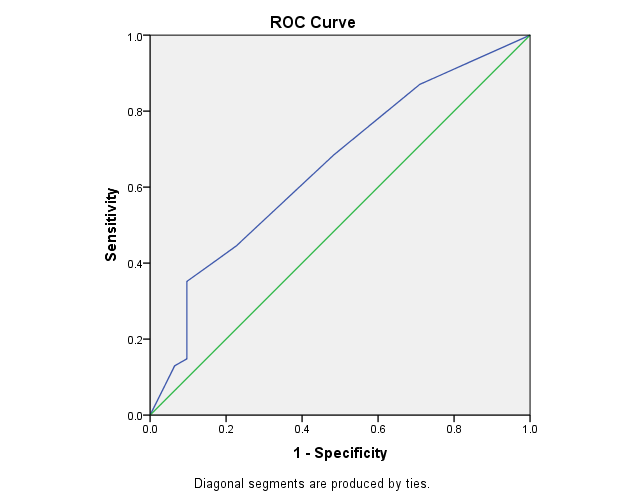   1. Joining support program |

Supplementary Figure 1. Receiver operating characteristic (ROC) curve exploring the predictive validity of the Generalized Anxiety Disorder 2-item scale (GAD-2) among the mothers of children with intellectual disabilities.
