# Supplementary material for: The Arabic Generalized Anxiety Disorder 2 (GAD-2): Psychometric evaluation among mothers of children with intellectual disabilities
Source: Dialogues Clin Neurosci. 2026 May 5;28(1):21–31. doi: 10.1080/19585969.2026.2650296 (PMC13148081; doi:10.1080/19585969.2026.2650296)
Supplement: Supplementary_Table1_GAD_2.docx [file TDCN_A_2650296_SM7250.docx]

**Supplementary Table 1.**

Quartile scores and categories of variables used as outcome variables in ROC analysis (N = 85)

| **Characteristics** | **Median (Q1-Q3)** | | **Categories** | |
| --- | --- | --- | --- | --- |
| Mood | | 6 (4-8) | ≥4 (good mood) | <4 (low mood) ● |
| Sleep quality | | 5 (4-7) | ≥4 (good sleep quality) | <4 (poor sleep quality) ● |
| Perceived physical health | | 3 (3-4) | ≥3 (good physical health) | <3 (poor physical health) ● |
| Stress | | 3 (2-5) | ≥5 (high stress) ● | <5 (low stress) |
| Happiness | | 6 (5-8) | ≥5 (high happiness) | <5 (low happiness) ● |

●: Target groups that possibly possess the condition being investigated by ROC analysis i.e., high anxiety
